# Chemical Communication between Heart Cells is Disrupted by Intracellular Renin and Angiotensin II: Implications for Heart Development and Disease

**DOI:** 10.3389/fendo.2015.00072

**Published:** 2015-05-19

**Authors:** Walmor C. De Mello

**Affiliations:** ^1^School of Medicine, University of Puerto Rico, San Juan, PR, USA

**Keywords:** chemical, communication, heart, cell, metabolic cooperation

## Abstract

**Highlights**
- Intracellular renin and angiotensin disrupts chemical communication in heart.
- Epigenetic modification of renin angiotensin aldosterone system (RAAS) and heart disease.
- Intracrine renin angiotensin and metabolic cooperation.
- Gap junction, intracellular renin and angiotensin, cellular patterns, and heart development.

The finding that intracellular renin and angiotensin II (Ang II) disrupts chemical communication and impairs metabolic cooperation between cardiomyocytes induced by aldosterone, hyperglycemia, and pathological conditions like myocardial ischemia is discussed. The hypothesis is presented that epigenetic changes of the renin angiotensin aldosterone system (RAAS) are responsible for cardiovascular abnormalities, including the expression of RAAS components inside cardiac myocytes (intracrine RAAS) with serious consequences including inhibition of electrical and chemical communication in the heart, resulting in metabolic disarrangement and cardiac arrhythmias. Moreover, the inhibition of gap junctional communication induced by intracellular Ang II or renin can contribute to the selection of cellular patterns during heart development.

## Gap Junctions and Cellular Patterns

Cells are functional units, which play a fundamental role on tissue and organ functions. The cellular arrangement established during embryonic development is governed by several genes, which regulate the properties of surface cell membrane including the expression of receptors and ligand molecules such as glycoproteins which make possible the recognition and the interaction between cells. In this process, chemical communication between cells play an important role making possible the establishment of cellular patterns, which are essential for tissue and organ development (1, 2). The synthesis of intercellular channels (gap junctions) contributes to the metabolic cooperation between cells through the spread of ions and small molecules such as amino acids, hormones, and nucleotides from cell-to-cell (3). Recently, evidence has been presented that much larger molecules such as peptides and microRNA are able to diffuse through gap junctions (4, 5). Because microRNA-133a engineered mesenchymal stem cells augment cardiac function and cell survival in the infarct heart (6), it is conceivable that the transfer of larger molecules through gap junctions represents an important aspect of metabolic cooperation in health and disease in part by modulating renin angiotensin aldosterone system (RAAS) (7). Indeed, novel evidence that miRNAs are important regulators of biological processes involved in cardiovascular disease via genetic control is now available (7).

The gap junction permeability is modulated by different factors such as intracellular Ca concentration and cyclic AMP, which enhances the permeability of intercellular channels through the phosphorylation of the gap junction proteins (connexins) (8, 9). Recently, it was found that high glucose inhibits chemical communication between heart cells including the intercellular flow of glucose (10) –an effect partially explained by hyperphosphorylation of gap junction proteins but also due to intracellular Ang II generated by high glucose (10). The evidence now available that glucose flows from cell-to-cell through gap junctions (11) indicates that the exchange of energy substrate between cells is an important mechanism of tissue homeostasis, particularly during ischemic conditions when glucose becomes an important source of energy.

## Angiotensin II, Gap Junctions, and Epigenetic Factors

The transfer of chemical signals between cells seems to play a decisive role during tissue regeneration (3), and more recently, evidence has been provided that misregulation of the gap junction protein connexin43 (Cx43), when DNA sequence of the Cx gene itself is unaltered (12), changes cardiogenesis. Over the past few years, important knowledge about the epigenetic regulation of heart development and disease has been achieved. It is known, for instance, that DNA methylation, chromatin remodeling, and histone modifications are involved in this process (13), and that the reactivation of fetal genes are involved in the development of heart failure and ventricular hypertrophy. It is also known that different epigenetic factors like electric fields and pressure alter cardiac morphology and functions through gap junctions (12), and that there is an association between fetal insults to epigenetic changes of genes with consequent generation of pathological processes including hypertension (14). Evidence is available that in maternal low protein diet rat models of programing, angiotensin converting enzyme inhibitors or angiotensin receptor antagonists administered early in life can prevent development of hypertension (14). It is then reasonable to think that cardiac abnormalities can be generated by the modification of the RAAS elicited by epigenetic factors. During early stages of embryonic development, a large proportion of genes is demethylated, but during development a selective methylation of unnecessary genes occurs in the differentiated cell (14). Moreover, post translational changes of calcium handling proteins, such as calstabin2, induced by epigenetic factors can alter cardiac structure. Histological studies of the heart revealed that aged Calstabin2 null mice exhibited large areas of cell death myocardial fibrosis (15).

Because Ang II reduces the gap junction permeability, the peptide can play a role during development impairing the transference of chemical information between cardiac cells and contributing to the selection of cellular patterns, which are essential during embryonic development (1, 2). It is known that ACE and other components of the renin angiotensin system are expressed in the embryo after 30 days of gestation (16), and that Ang II plays a role on the differentiation of embryonic stem cells into smooth muscle cells (17) –an effect mediated by AT1 receptor (18). In humans, AT1 and AT2 receptors are expressed early during embryonic development (24 days of gestation) (19).Furthermore, stress, *in utero*, can induce the later development of disease including the overexpression of AT1b receptors in the adrenals and hypertension (14).

## RAAS, Metabolic Cooperation, and Organogenesis

Evidence provided in recent decades revealed that local expression of renin angiotensin or aldosterone impairs communication between heart cells by altering the gap junction conductance (20–24). Angiotensin II, for instance, plays an important role on the modulation of cell communication, inward calcium current, heart contractility, and cell volume regulation in health and disease through activation of cell surface AT1 receptors as well as PKC or tyrosine kynases (22, 24, 25). The role of gap junctions, however, is not limited to the spread of electrical currents between cardiac cells, but is also involved in the exchange of chemical signals including amino acids, nucleotides, and molecules up to 1000 Da, which can flow from cell-to-cell through gap junctions (9). This means that cardiac cells exchange important metabolites, which are vital for tissue function. The recent finding that intracellular renin and angiotensin II disrupts cell communication in the heart, and is involved in cell volume regulation (25–27), indicates that the presence of RAAS components inside the cardiac cell (intracrine effect) has an important functional significance (22), particularly during pathological conditions like cardiac failure, when the heart tends to return to embryological conditions and organogenesis is possible (28).

## On the Possible Role of the Intracrine RAAS

The presence of RAAS inside the cells, and the functional role of intracellular renin and Ang II on the regulation of cell communication and inward calcium current lead to the concept of an intracrine RAAS (11, 24, 29–32).The hypothesis outlined here is that the intracrine renin–angiotensin system activated by aldosterone (21), hyperglycemia (33, 34), or by pathological conditions like heart failure and myocardial ischemia [when a renin transcript is overexpressed (35, 36)] is part of the genetic makeup, which is known to occur during these pathological conditions. It is feasible that during pathological conditions, a drastic change of intercellular chemical and electrical communication occurs, involving second messengers and other signal molecules, which are implicated in cell proliferation and growth. Supporting this idea is the finding that angiotensin II, which is a growth factor, causes electrical uncoupling in the failing heart through the activation of AT1 receptors and intracellular pathways, such as PKC, MAPK family, and increment of intracellular calcium (24), and causes disruption of chemical communication between heart cells (28). Similar effect was found when renin was dialyzed inside the heart cells – an effect not necessarily related to Ang II formation (37), but in part related to oxidative stress probably induced by the activation of an intracellular renin receptor elicited by renin (37, 38).

**Figure 1:**
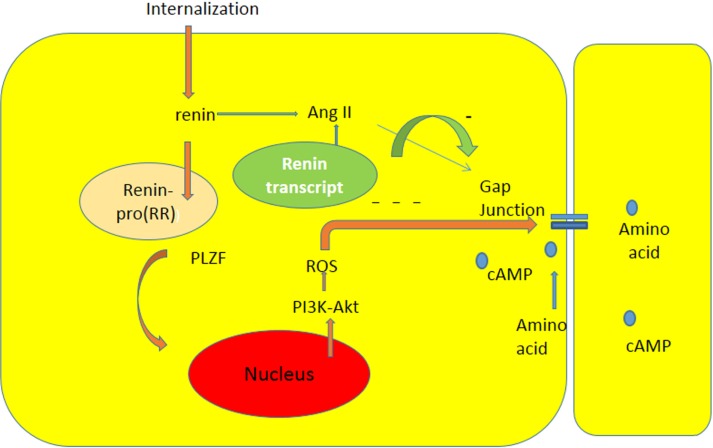
**Diagram illustrating the influence of intracellular renin due to internalization or to expression of renin transcript on chemical communication between heart cells**. The interaction between renin and pro (renin) receptor occurring inside a cytoplasmic vesicle can induce: the formation of Ang II, and the translocation of PLZF to the nucleus [see Ref. (38)], and consequent generation of oxidative stress through PI3K-Akt pathway with consequent decline of gap junction communication.

Studies of Schefe et al. (38) indicated that there is an intracellular renin receptor, which when activated by intracellular renin, causes the translocation of a transcription factor (PLZF) from the cytoplasm to the nucleus, and consequent activation of several genes including an enhanced transcription of the p85a subunit of the phosphatidylinositol-3kinase(PI3K–p85a).Furthermore, other studies (39) revealed that PI3K-Akt signaling activates production of ROS by opening of the mitochondrial ATP-sensitive K channel (mKATP channel). It is reasonable to think that the activation of pro (renin) receptor by renin within an intracellular vesicle increases the oxidative stress through the PI3-Akt pathway with consequent decrease of gap junction permeability [see Ref. (37)] (see Figure 1).This finding is of particular interest because previous studies revealed the presence of renin transcripts, which encode a cytosolic protein that cannot be secreted (35). According to these studies, these transcripts, which are derived from the same renin gene using another promoter located within intron A, are overexpressed during myocardial infarction (35), suggesting a possible functional role of the enzyme. These isoforms of renin are present in mitochondria, and are generated at free ribosomes in the cytoplasm (36). It is known that different components of the RAAS can be transported intracellularly via secretory vesicles to mitochondria or to the nucleus, and that the activation of the mitochondrial Ang system is coupled to mitochondrial nitric oxide production. Furthermore, the binding of Ang II to mitochondrial AT2 receptors stimulates NO formation with consequent suppression of mitochondrial oxygen consumption (40). Considering the role of Ang II on oxidative stress, it is reasonable to think that the activation of AT1 or AT2 receptors in mitochondria might be involved in the etiology of cardiovascular disease.

## ACE/Ang II/AT1 Receptor Axis Versus ACE2/Ang(1-7)/Mas Receptor Axis

Evidence is now available that the formation of angiotensin (1–3, 8, 9, 12, 20) [Ang (1–7) elicited by the activation of ACE2 (39)] has beneficial effects in the heart (39, 41, 42), counteracting the harmful effects of Ang II (43) and re-establishing impulse conduction during myocardial ischemia (42) as well as after cell swelling (44). It is then conceivable that Ang (1–7) can play a role in the formation of functional cellular patterns in the adult heart by enhancing gap junction permeability with consequent improvement of metabolic cooperation between cardiac cells. The mechanism by which Ang (1–7) enhances gap junction permeability involves activation of PKA and phosphorylation of gap junction channels (44).

The possible role of ACE2/Ang (1–7) axis activation during embryological development cannot be discarded because recent studies revealed that ACE2 contributes to the postnatal development of the heart, enhances coronary artery remodeling, and causes cardiac hypertrophy (45, 46). In the ACE2 knockout animal model, for instance, a mild left ventricular wall thinning, ventricular dilation, and reduction of heart contractility as well as, and gene expression of the hypoxia-genes have been described (45, 46).

**Figure 2:**
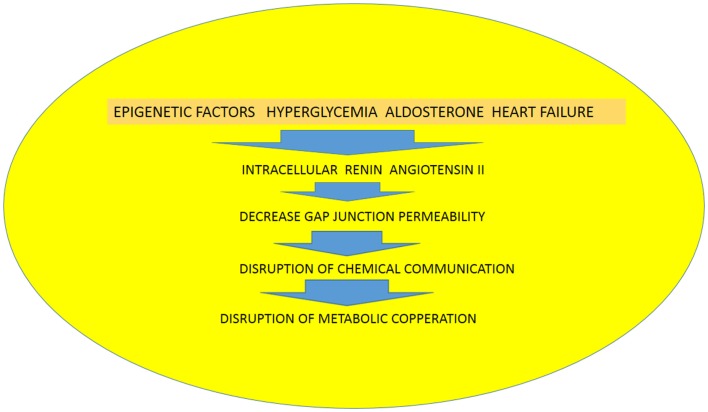
**Diagram illustrating the role of epigenetic factors, hyperglycemia, aldosterone and heart failure on the expression of renin and angiotensin II in heart cells with consequent disruption of chemical communication and metabolic cooperation**.

Recently, Ang (1–7) has been found in the nuclei of NRK-52E renal epithelial cells (47), but the precise meaning of this finding is not known. The possibility that the heptapeptide be internalized cannot be discarded because internalization of Mas receptors through a clathrin-mediated pathway has been described when activated by Ang (1–7) (48).Because it is known that gap junctions are synthesized within minutes (29, 49–51) (half-life <5 h) followed by their return into the cell (51) utilizing the endocytic clathrin machinery (52), it is plausible to assume that the establishment of functional cellular pathways, which is a dynamic process, can be generated by the activation of the ACE2/Ang (1-7)/Mas receptor axis. The activation of the ACE/Ang II/AT1receptor axis, on the other hand, with consequent formation of angiotensin II and aldosterone (21), disrupts the exchange of chemical signals between heart cells and contributes to the rupture of functional cellular patterns with consequent inhibition of metabolic cooperation between cardiac cells. The increment of oxidative stress seems to be an important cause of disruption of cell communication (10, 37), and evidence is available that Ang II receptors are involved in this process. The binding of Ang II to mitochondria AT2 receptors (mtAT2Rs), for instance, stimulates NO formation with consequent suppression of mitochondrial oxygen consumption (40), with serious consequences for heart physiology. Considering the role of Ang II on oxidative stress, it is reasonable to think that activation AT1or AT2 receptors in mitochondria might be involved in the etiology of heart disease including heart failure. It is known, for instance, that AT1R blockade decreases RAS-mediated activation of NADPH oxidase and oxidative stress, reducing left ventricular fibrosis and mitochondrial remodeling (53). According to our hypothesis, epigenetic factors as well as hyperglycemia, aldosterone, and heart failure, or ischemia lead to the expression of renin and Ang II inside the cardiac cells with consequent decrease of gap junction permeability, disruption of chemical communication, and consequent impairment of metabolic cooperation, resulting in heart tissue dysfunction (see Figure 2). Furthermore, the balance between ACE/Ang II/AT1 receptor axis and the ACE2/Ang(1-7)/Mas receptor axis may play an essential role on the formation of cellular patterns during embryological development, but also in the differentiated heart by altering the gap junction permeability and metabolic cooperation between cells (see Figure 1).

## Perspective

Future studies on the influence of intracellular renin and Ang II on the interchange of chemical signals between cardiac cells will provide a better knowledge of how metabolic cooperation contributes to heart physiology and disease and will help the development of novel therapeutic approaches for cardiac diseases.

## Conclusion

(1) Intracellular renin disrupts the chemical communication between heart cells with serious consequences for metabolic cooperation and electrical synchronization in the heart. The finding that there is a renin transcript overexpressed during myocardial infarction might indicate that an increase of renin inside the heart cell is an important cause of functional disarrangement; (2) gap junctions play an important role on metabolic cooperation between heart cells and are involved in the establishment of cellular patterns during heart development as well as in the adult heart; (3) the hypothesis is presented that epigenetic changes of the RAAS are responsible for hypertension and cardiovascular abnormalities including the expression of RAAS components inside cardiac myocytes (intracrine RAAS) (see Figure 2); (4) diseases like hypertension, heart failure, and myocardial ischemia induce the synthesis of RAAS components in heart cells and causes severe abnormalities of tissue functioning; (5) high glucose inhibits the chemical communication between heart cells with serious implications for the diabetic heart.

## Conflict of Interest Statement

The author declares that the research was conducted in the absence of any commercial or financial relationships that could be construed as a potential conflict of interest.
